# Occipital Screw Placement Using a Navigation System for a Pathological Odontoid Fracture With a Dural Venous Sinus Variation

**DOI:** 10.7759/cureus.16610

**Published:** 2021-07-25

**Authors:** Kousei Miura, Masao Koda, Toru Funayama, Hiroshi Takahashi, Masashi Yamazaki

**Affiliations:** 1 Department of Orthopaedic Surgery, Faculty of Medicine, University of Tsukuba, Tsukuba, JPN

**Keywords:** occipito-cervical fusion surgery, navigation, occipital sinus, occipital screw, variation

## Abstract

Conventional fluoroscopic guidance can provide enough information to precisely insert an occipital screw in ordinary cases. However, the occipital screw creates a potential risk of dural venous sinus injury or thrombosis. In some cases, with dural sinus variation, surgeons must especially be cautious to avoid its injury. We present a rare case of proper occipital screw placement using a navigation system for a pathological odontoid fracture with a high risk of dural venous sinus injury because of anatomical variations in the transverse and occipital sinuses. A 60-year-old man who underwent thyroidectomy at the age of 37 years for thyroid carcinoma developed acute neck pain and quadriparesis due to falling out of bed. He urgently underwent closed reduction and temporary immobilization with a halo-vest for a pathological odontoid fracture and atlantoaxial dislocation. Preoperative contrast-enhanced CT showed an absent right transverse sinus and a prominent occipital sinus as variations of the dural venous sinuses. Occipito-C7 fusion surgery was performed without intraoperative active venous bleeding or postoperative brain disorder by using a navigation system for the occipital screw placement to avoid injury to the dural sinus. Postoperative computed tomography showed bi-cortical occipital screw placement avoiding the prominent occipital sinus. The patient’s postoperative course was uneventful. In this case, although rigid occipito-cervical fixation using bi-cortical occipital screws was needed for the pathological odontoid fracture, the variation of the occipital sinus created a high risk of injury during occipital screw placement with conventional fluoroscopic guidance. There is an anatomical variation of the dural venous sinuses between individuals. Prominent occipital sinus injury may notably cause fatal complications such as massive bleeding or occlusion. Thus, we safely inserted the occipital screws using a navigation system that enabled us to avoid occipital venous sinus injury. Occipital screw placement with a navigation system can be a better option to prevent dural venous sinus injury in cases where there is variation in the dural venous sinuses, such as with a prominent occipital venous sinus.

## Introduction

Recent advances in navigation systems for spinal fusion surgery have increased the safety of screw insertion. Navigated pedicle screw insertion has greater accuracy compared to non-navigated insertion for all spinal regions [1,2]. However, our literature search revealed no reports of occipital screw placement using navigation systems for occipito-cervical fusion surgery. That is because conventional fluoroscopic guidance can provide enough information in ordinary cases to precisely insert the occipital screw. However, the occipital screw has potential risks such as dural injury, cerebrospinal fluid (CSF) leakage, dural venous sinus injury or thrombosis, and sub- or epidural hematoma [3]. When variations in the dural venous sinuses such as the occipital sinuses are present, surgeons should be cautious to avoid their injury [4]. Fluoroscopic guidance may be insufficient in that case. Here, we present the case of proper occipital screw placement using a navigation system for a pathological odontoid fracture with a high risk of dural venous sinus injury because of its anatomical variation.

## Case presentation

**Figure 1:**
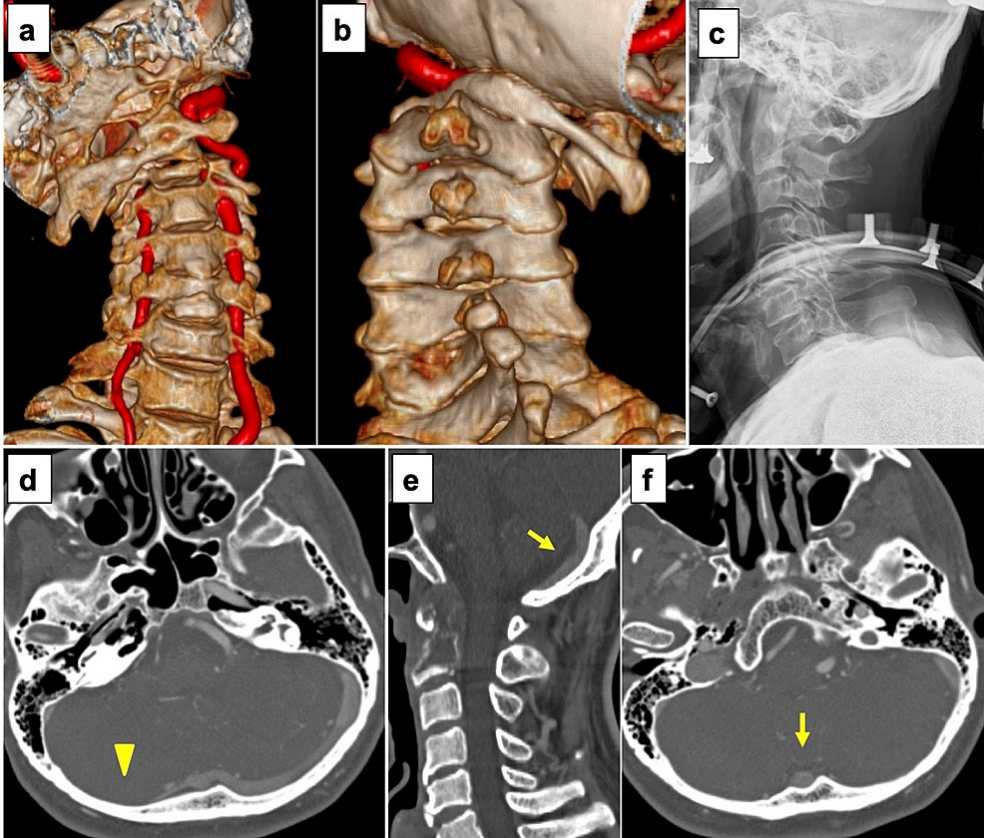
Preoperative images (a, b) Three-dimensional reconstruction of computed tomography (CT) image showing a pathological odontoid fracture and atlantoaxial dislocation. (c) Lateral radiograph showing reposition after closed reduction with a halo-vest. (d) Contrast-enhanced axial CT showing an absent right transverse sinus (arrowhead). (e), (f) Contrast-enhanced CT showing a prominent occipital venous sinus (arrows).

A 60-year-old man underwent thyroidectomy at the age of 37 years for thyroid carcinoma. He was found to have metastases to the odontoid process and multiple lung metastases on positron emission tomography/computed tomography (PET/CT) one month before surgery. Lenvatinib and radiation therapy were planned because he initially had no neck pain. However, he developed acute neck pain and quadriparesis after falling from bed and was urgently referred to our department. The patient, whose preoperative neurologic loss was graded at modified Frankel C1, exhibited neck rotatory deformity and severe muscle weakness (manual muscle testing [MMT], 1-2 level) of the upper and lower limbs bilaterally. CT revealed a pathological odontoid fracture and atlantoaxial dislocation. He urgently underwent closed reduction and temporary immobilization with a halo-vest under general anesthesia and was scheduled to undergo occipito-cervical fusion surgery for definite stabilization. Contrast-enhanced CT after closed reduction showed an absent right transverse sinus and a prominent occipital venous sinus (Figure 1).

**Figure 2:**
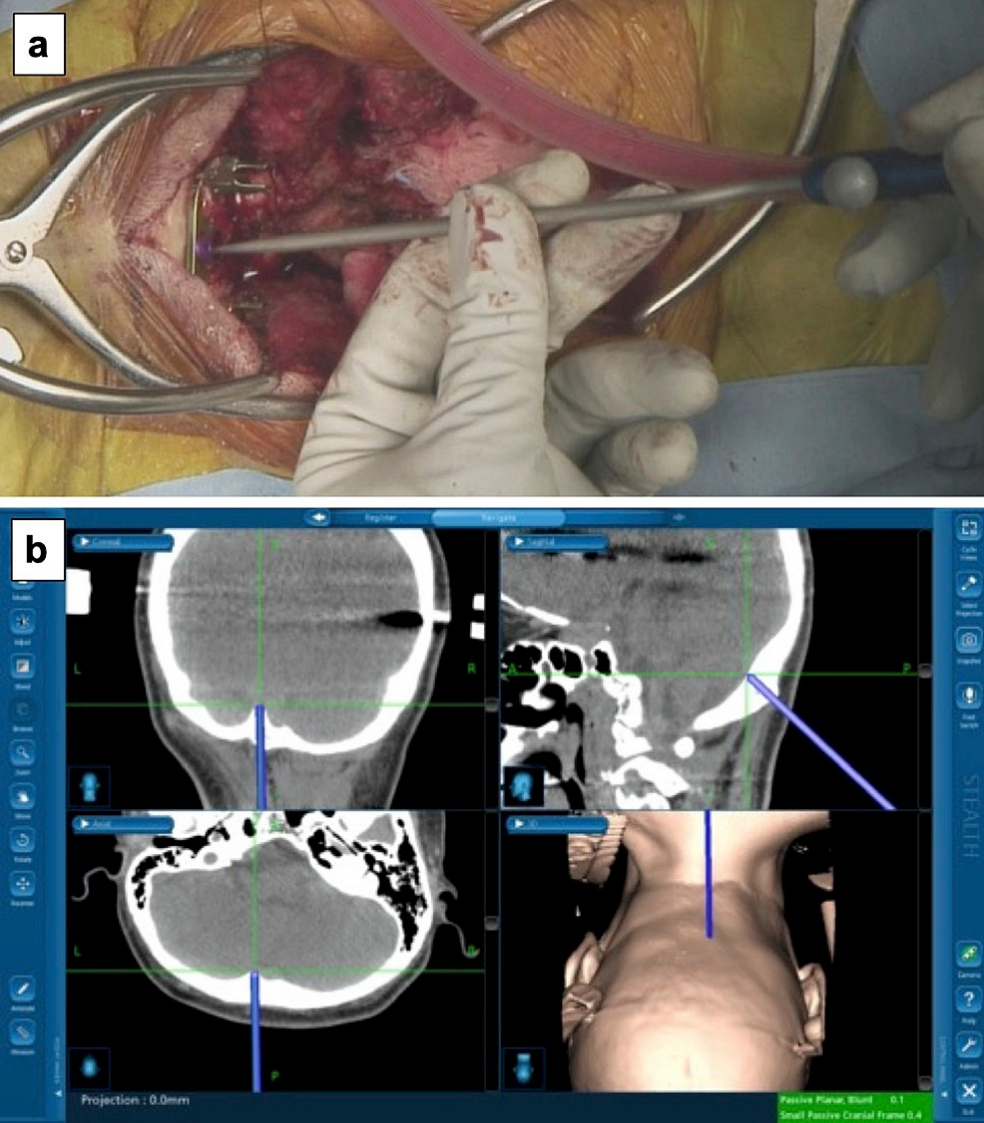
Intraoperative navigation for inserting occipital screws (a) An intraoperative view of placement of the navigation probe at the screw hole of the occipital plate. (b) A screenshot of the intraoperative navigation system showing multiple views of the position of the navigation probe.

Occipito-C7 fusion surgery was performed without intraoperative active venous bleeding or postoperative brain disorder. We inserted the occipital screws using a navigation system (StealthStation S8; Medtronic Inc, Minneapolis, MN, USA) due to variation of the occipital venous sinus that could cause its injury during occipital screw placement (Figure 2). Preoperative CT data were transferred to the navigations system workstation. The patient was placed in the prone position with the Mayfield skull clamp under general anesthesia. The reference arm was attached to the Mayfield clamp, and registration was performed by tracing the head surface with a pointer. After exposure from the occipital bone to C7, we inserted the occipital screw with fluoroscopy. During placement of the occipital screw, the accuracy of occipital screw trajectory was confirmed by using a pointer of the navigation system to avoid the occipital venous sinus injury. The rod was fixed following cervical screw placement with fluoroscopy.

**Figure 3:**
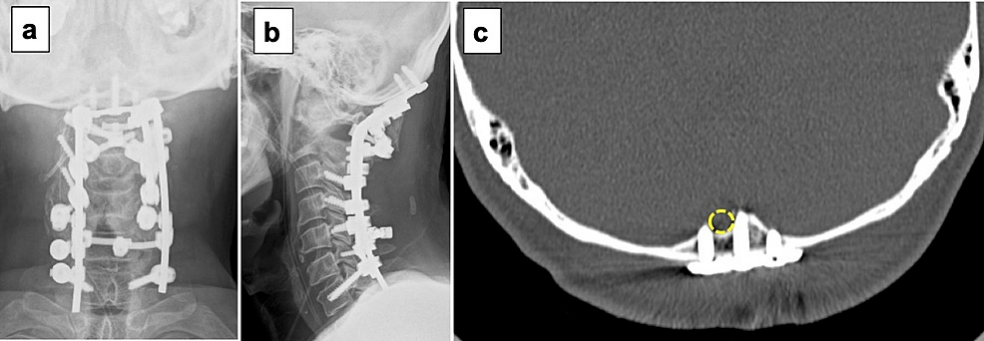
Postoperative images (a, b) Postoperative radiographs showing posterior occipito-C7 fusion. (c) Postoperative axial CT showing bi-cortical occipital screw placement avoiding the prominent occipital venous sinus (dotted circle) and no intracranial hematoma.

Postoperative CT showed bi-cortical occipital screw placement avoiding the prominent occipital venous sinus and no intracranial hematoma (Figure 3).

His motor weakness improved to the MMT 4-5 level, and he could walk with one crutch six months after surgery.

## Discussion

In this case, occipital screw placement using a navigation system during occipito-cervical fusion surgery avoided injury to the occipital venous sinus that could have been fatal. Occipito-cervical fusion surgery is usually performed for high cervical instability [5]. Implant failure such as screw loosening and pullout in occipito-cervical fusion surgery tends to occur at the occipital part [3]. Thus, the pullout strength of the occipital screw must be high to prevent failure. Many approaches have been investigated to evaluate the strength of the occipital screw using biomechanical analysis [6-8]. The bi-cortical occipital screws have 50% greater strength than uni-cortical screws [6]. The pullout strength of occipital screws is related to the thickness of the occipital bone, which is thicker at the midline than laterally. Accordingly, midline insertion of bi-cortical screws is the ideal placement [7,8].

On the other hand, it has been recognized that there are some possible risks such as dural injury, CSF leakage, dural venous sinus injury or thrombosis, and sub- or epidural hematoma during occipital screw placement [3]. Nadim et al. reported in a cadaveric study that occipital screws should be inserted at least 2 cm below the superior nuchal line to avoid dural venous sinus injury [9]. However, Izeki et al. found that the dural venous sinus is extremely variable between individuals, based on CT venography, so that there is no universal position for safe occipital screw placement that can apply to all individuals [10]. In particular, the variation of the occipital venous sinus located at the midline of the internal occipital bone should be considered carefully during occipital screw placement [4]. A prominent occipital venous sinus, as shown in this case, is the main drainage for the deep cerebral veins, and its injury may cause fatal complications such as massive bleeding or occlusion [11]. 

In this case, the absent right transverse sinus and the prominent occipital venous sinus as an idiopathic anatomic variation were found on preoperative contrast-enhanced CT. Although rigid occipito-cervical fixation using bi-cortical occipital screws was needed for this pathological odontoid fracture, the variation of the occipital venous sinus created a high risk of its injury during occipital screw placement with conventional fluoroscopic guidance. Thus, we safely inserted the occipital screws using a navigation system that enabled us to avoid injury to the occipital venous sinus. The postoperative course was uneventful without any implant failure. In ordinary cases, conventional fluoroscopic guidance can be enough to precisely insert the occipital screw because navigation needs extra time for registration. However, using a navigation system may be a safe option for variations of the occipital venous sinus, possibly preventing injury during occipital screw placement.

## Conclusions

There is a great anatomical variation of the dural venous sinuses between individuals. Variations of the dural venous sinus, such as a prominent occipital venous sinus, require attention to the location of the occipital screw. Prominent occipital sinus injury may notably cause fatal complications such as massive bleeding or occlusion. Occipital screw placement with a navigation system can be a better option to avoid dural venous sinus injury in those cases.
